# Identification of an altered gut microbiome and the protective effect of microbiome changer in prion diseases

**DOI:** 10.1186/s13567-025-01699-2

**Published:** 2026-01-17

**Authors:** Yong-Chan Kim, Sae-Young Won, Byung-Hoon Jeong

**Affiliations:** 1 Department of Biological Sciences, Andong National University, Andong, 36729 Republic of Korea; 2 School of Life Sciences and Biotechnology, Gyeongkuk National University, Andong, 36729 Republic of Korea; 3 Korea Zoonosis Research Institute, Jeonbuk National University, 820-120, Hana-Ro, Iksan, Jeonbuk 54531 Republic of Korea; 4 Department of Bioactive Material Sciences and Institute for Molecular Biology and Genetics, Jeonbuk National University, Jeonju, Jeonbuk 54896 Republic of Korea

**Keywords:** Prion, scrapie, metagenomics, microbiome, gut–brain axis, EGCG, drug

## Abstract

Prion diseases are fatal and contagious brain disorders caused by a pathogenic prion protein (PrP^Sc^) derived from the benign prion protein (PrP^C^). To date, there are no therapeutic substances to completely block prion diseases. Thus, the development of a therapeutic substance is necessary, and the identification of a novel biomarker of prion disease is the first essential step to develop new drugs. In the present study, we carried out a metagenomic analysis to identify microbiome biomarkers for prion disease using next-generation sequencing and bioinformatics tools in intraperitoneally prion-infected mice. In addition, we evaluated the protective effects of epigallocatechin-3-gallate (EGCG), a potent microbiome changer, in prion-infected mice by western blotting and survival analysis. We found a total of 14 differentially abundant taxa between prion-infected and control mice. In addition, we found that prion diseases caused altered microbiome networks and upregulation of DNA repair-related pathways. Furthermore, we observed the protective effect of the microbiome changer EGCG against prion disease in prion-infected mice. Given previous reports of microbiome alterations in prion diseases, we further validated these associations and demonstrated the protective effects of a microbiome-modulating compound.

**Supplementary Information:**

The online version contains supplementary material available at 10.1186/s13567-025-01699-2.

## Introduction

Prion diseases are fatal, chronic, and infectious neurodegenerative encephalopathies caused by an aggregated prion protein (PrP^Sc^), which is converted from the normal prion protein (PrP^C^) [1–6]. Although several attempts have been made to develop a therapeutic substance for prion disease, there are no suitable therapeutic substances to cure prion diseases [7–10]. Thus, the development of a therapeutic substance is important, and the identification of a novel biomarker of prion diseases is the first essential step to develop new drugs.

Although the major lesion site of prion infection is the brain, PrP^Sc^ has also been detected in peripheral tissues, including gastrointestinal organs, skin, and bone marrow [11–14]. Conversely, physiological factors derived from peripheral tissues, such as the replication and accumulation of PrP^Sc^ in lymphoid organs, may influence the progression of prion diseases by facilitating the spread of prions to the central nervous system through peripheral nerves [15, 16]. Similarly, recent studies have reported that the gut–brain axis, the bidirectional network between the central and enteric nervous systems, plays a pivotal role in the physiological function and pathology of the brain [17–19]. Recent studies in prion-infected mice and patients with Creutzfeldt–Jakob disease (CJD) have revealed distinct gut microbiome alterations associated with changes in short-chain fatty acid metabolism and neuroinflammation, highlighting the importance of validating the microbiome–prion disease association [20, 21]. Since prion diseases have the gastrointestinal tract as the main route of infection [16] and the gut microbiome can also affect the pathophysiology of prion diseases in the brain via the gut–brain axis, validation of the association between the microbiome and prion diseases is important to investigate the possibility of the microbiome as a drug target for prion diseases.

In the present study, we intraperitoneally inoculated phosphate-buffered saline (PBS) and ME7 scrapie into mice and sacrificed them at the end stage of prion diseases to collect fecal metagenomic DNA. In addition, we performed metagenomic analysis to identify microbiome biomarkers for prion disease using next-generation sequencing and bioinformatics tools. Furthermore, we assessed the protective effects of epigallocatechin-3-gallate (EGCG), a potent microbiota changer, in prion-infected mice by western blotting and survival analysis [22, 23].

## Materials and methods

### Ethical statements

C57BL/6J mice were provided by Nara Biotech (Pyeongtaek, Korea). All experiments were approved by the Institute of Animal Care and Use Committee (IACUC) of Jeonbuk National University (JBNU 2021-0144).

### Animals

For the metagenomic analysis, six female mice were used, consisting of three control (CTL) mice and three ME7-inoculated mice. The ME7 prion strain was administered via intraperitoneal injection. These animals were sacrificed at 7 months post inoculation, upon the onset of clinical symptoms such as shuffling gait, reduced locomotor activity, hindlimb weakness, and difficulty in maintaining balance. Both brain and fecal samples were collected.

To evaluate the therapeutic efficacy of EGCG, a total of 19 female mice were used: 3 control (CTL) mice, 8 ME7-inoculated mice, and 8 ME7-inoculated mice treated with EGCG. At 5 months post inoculation, three mice from each group were euthanized for western blot analysis, while the remaining five ME7-inoculated mice and five ME7-inoculated mice treated with EGCG were subjected to survival analysis.

All mice were housed under identical conditions with a 12-h light/12-h dark cycle, at  20–22°C with 40–60% humidity, and were fed the same standard laboratory diet throughout the study period. All mice were euthanized by cervical dislocation under inhalation anesthesia, in accordance with IACUC guidelines.

### Western blotting

Brain tissue samples were homogenized in RIPA buffer (Thermo Fisher Scientific, Waltham, USA) containing a proteinase inhibitor cocktail (Roche, Munich, Germany). Protein concentrations were measured using the Bradford assay, employing Protein Assay Dye Reagent Concentrate (Bio-Rad, Hercules, CA, USA), according to the manufacturer’s instructions. The quantified samples were boiled to 95 °C for 10 min in sodium dodecyl sulfate (SDS) sample loading buffer (Thermo Fisher Scientific, Waltham, USA) and loaded in a 10–15% SDS gel. The proteins were transferred to a nitrocellulose membrane (Amersham, Little Chalfont, UK) at 100 V for 1.5 h, and the membranes were blocked with Tris-buffered saline with Tween-20 (TBST) (Thermo Fisher Scientific, Waltham, USA) containing 5% skim milk (Santa Cruz Biotechnology, Dallas, USA) for 1.5 h. The membranes were incubated for 12 h with primary antibodies. For western blot analysis, the following primary antibodies were used: anti-PrP monoclonal antibody SAF84 (Bertin, Montigny-le-Bretonneux, France) with 1:200 dilution, anti-GFAP antibody (2E1) (Santa Cruz, Dallas, TX, USA) with 1:100 dilution, and anti-HSP90 antibody (BD Transduction Laboratories, San Jose, CA, USA) with 1:200 dilution. All antibodies were raised against mouse targets. The SAF84 antibody was employed to detect PrP accumulation, GFAP was utilized to evaluate astrocytosis, and HSP90 served as a housekeeping control to confirm equal protein loading across samples. The membranes were incubated with horseradish peroxidase-conjugated secondary antibodies (Sigma-Aldrich, St. Louis, USA) for 1 h. Then, the proteins were detected by a Pierce ECL kit (Thermo Fisher Scientific, Waltham, USA).

### PrP^Sc^ detection

To detect PrP^Sc^ in the brains of prion-infected mice, 50 µg/mL proteinase K was added to quantified brain homogenate for 1 h at 37 °C. The proteinase K-added samples were boiled to 95 °C for 10 min in SDS sample loading buffer (Thermo Fisher Scientific, Waltham, USA). The proteinase K-added samples were used to visualize the PrP^Sc^ bands by western blotting.

### Feces collection

Fecal samples were collected on the same day from both control and ME7-inoculated mice at 7 months post inoculation, immediately after the animals were sacrificed. Fresh feces were collected into individual centrifuge tubes. The samples were frozen at −80 °C until DNA extraction.

### Metagenomic analysis

The fecal metagenomic DNA was extracted from six fecal samples (three from PBS-inoculated mice and three from ME7 scrapie-inoculated mice) using the PureLink™ Microbiome DNA Purification Kit (ThermoFisher, NY, USA). The gut microbiota was determined with V3–V4 hypervariable regions of 16S ribosomal RNA (rRNA) gene sequencing using Illumina MiSeq sequencing (Illumina, CA, USA) with primers 341F and 805R, as described previously [24, 25]. The chimeric and low-quality reads were filtered out using the DADA2 program ver. 1.10. After merging paired-end reads, representative sequences were selected by removing duplicates, and the sampling depth was normalized to 40 566 sequences per sample using the minimum frequency method. For 16S rRNA gene sequencing, diversity and taxonomy were analyzed by the QIIME2 tool. Operational taxonomic unit (OTU) clustering was performed at 97% sequence similarity. Alpha diversity metrics, including observed OTUs, Shannon diversity, Pielou’s evenness, and Faith’s phylogenetic diversity, were calculated. Significant statistical differences between the gut microbiota of the prion-infected and control groups were analyzed by Wilcoxon rank-sum test. *P*-values were adjusted using false discovery rate (FDR) correction. Beta diversity was analyzed using a weighted UniFrac phylogenetic distance matrix, and a principal coordinates analysis plot was drawn. Taxonomy assignment was performed by supervised learning using the naïve Bayes algorithm against the Silva database with 99% similarity. The top 20 abundant taxa were visualized by bar plot. The edgeR program was used to analyze differentially abundant microbiomes (adjusted *P* < 0.01). The SCNIC program was used for correlation network analysis of the top 50 abundant microbiomes. The PICRUSt2 program was used for KEGG orthology (KO) analysis. The ALDEx2 program was used to identify distinct KO assignments between prion-infected and control mice. The ClusterProfiler program was used for gene set enrichment analysis (GSEA) of distinct KO assignments and to identify significantly enriched KEGG signaling pathways (adjusted *P* < 0.05).

### Evaluation of the protective effect of EGCG and survival analysis

The ME7 scrapie strain was provided by The Roslin Institute, The University of Edinburgh. C57BL/6 mice (6 weeks old) were intraperitoneally infected with 100 μL 1% (w/v) brain homogenate obtained from terminally ill ME7-infected mice. After 1 week, 100 μL EGCG (20 mg/kg) (Merck, Darmstadt, Germany) dissolved in 1X PBS was injected weekly to evaluate the protective effect of these materials against prion disease. At 5 months post injection, the mice were sacrificed and PrP^Sc^ accumulation and astrocytosis were investigated by western blotting. The relative quantification of the western blot bands was performed using ImageJ software (National Institutes of Health, Bethesda, Maryland, USA). The band intensity of each target protein was measured and normalized to the expression level of the housekeeping protein HSP90. The normalized values were then used to compare relative expression levels between experimental groups. Statistical analyses were conducted using SAS version 9.4 (SAS Institute Inc., USA). Three independent experiments were carried out. Statistical significance using the *P* value was calculated with a one-tailed Student’s *t* test for single comparisons. The symbols *, **, and *** indicate *P* < 0.05, *P* < 0.01, and *P* < 0.001, respectively.

To conduct survival analysis, the mice were observed daily until disease symptoms developed post injection and were then sacrificed. Brains were collected to detect PrP^Sc^ by western blotting. Kaplan‒Meier survival analysis was conducted by the survival and survminer packages of the R program. Statistical significance was evaluated using the log-rank test.

## Results

### Prion disease model construction and microbiome collection

**Figure 1:**
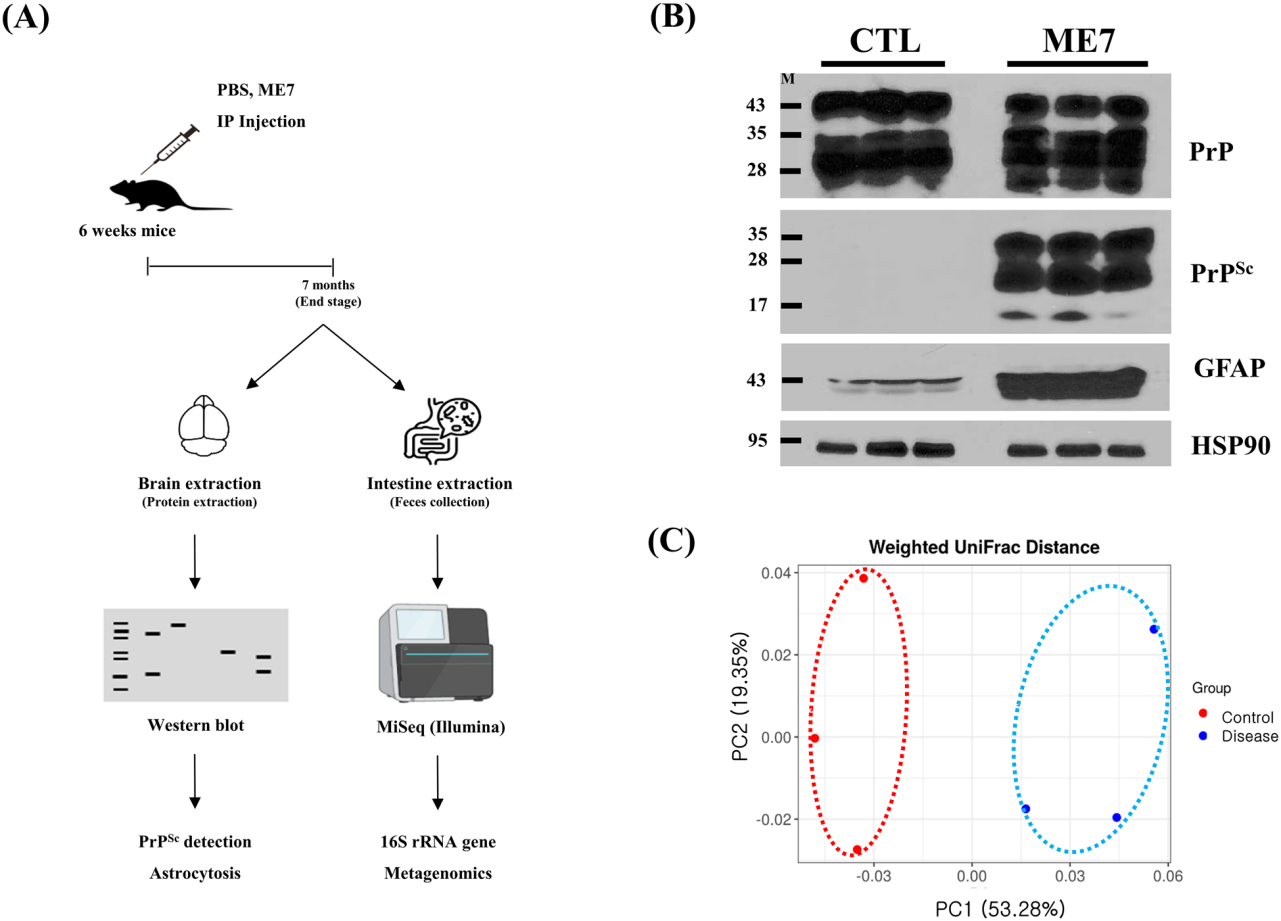
**Metagenomic analysis in prion-infected mice.** **A** The overall workflow for PrP^Sc^ detection and metagenomic analysis of prion-infected mice. IP, intraperitoneal. **B** Western blotting detection of PrP^Sc^ and astrocytosis markers in the brain. **C** Principal coordinates analysis plot of the statistical model for beta diversity of metagenomic data using weighted UniFrac phylogenetic distance matrix. Red dots indicate metagenomic data for the feces of C57BL/6 mice inoculated with PBS (CTL). Blue dots indicate metagenomic data for the feces of C57BL/6 mice inoculated with ME7 scrapie (ME7).

Detailed information on the workflow in the present study is shown in Figure 1A. In brief, we administered an intraperitoneal injection of the ME7 scrapie strain to 6-week-old mice and sacrificed the mice when disease symptoms developed. To diagnose prion diseases, we collected the brains of noninfected mice and prion-infected mice and carried out western blotting. As expected, PrP^Sc^ accumulation and astrocytosis were observed in the brains of prion-infected mice (Figure 1B). We collected feces to extract metagenomic DNA. The gut microbiota was annotated with hypervariable regions of the 16S rRNA gene using Illumina MiSeq sequencing. Principal coordinates analysis was conducted to evaluate the beta diversity of microbiota composition. Each group showed different and well-aligned clusters of microbiota composition (Figure 1C).

### Differentially abundant microbiomes between prion-infected and control mice

**Figure 2:**
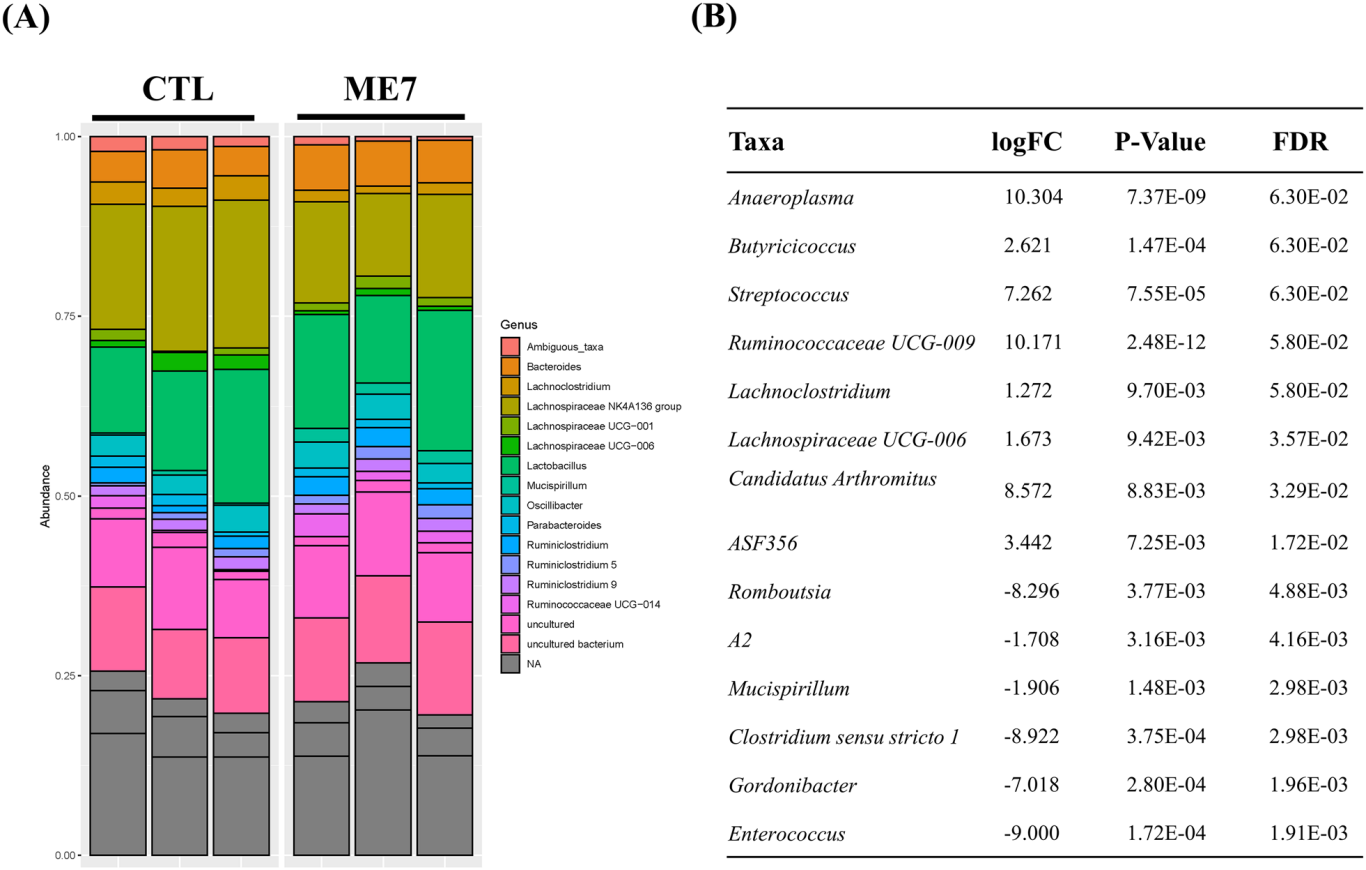
**Comparison of abundant microbiomes (genus level) between prion-infected and control mice.** **A** The top 20 abundant taxa in prion-infected and control mice. **B** Differentially abundant microbiomes between prion-infected and control mice (adjusted *P* < 0.01). A positive logFC number indicates a microbiome abundantly found in control mice. A negative logFC number indicates a microbiome found abundantly in prion-infected mice. FC: fold change, FDR: false discovery rate.

To illustrate the differences in the microbiota between prion-infected and control mice, we performed a bar plot analysis (Figure 2A). The top 20 abundant taxa roughly indicated that the relative abundance of different genera varied among the six fecal samples at the genus level. To find the significant differences in the microbiota between prion-infected and control mice, we carried out a differentially abundant microbiome analysis using the edgeR program (Figure  2B). A total of 14 differentially abundant taxa were identified. In brief, eight taxa, including *Anaeroplasma*, *Butyricicoccus*, *Streptococcus*, Ruminococcaceae UCG-009, *Lachnoclostridium*, Lachnospiraceae UCG-006, *Candidatus Arthromitus*, and ASF356, were abundant in control mice compared with prion-infected mice. In addition, six taxa, including *Romboutsia*, A2, *Mucispirillum*, *Clostridium* sensu stricto 1, *Gordonibacter*, and *Enterococcus*, were abundant in prion-infected mice compared with control mice. In the alpha diversity analysis, no significant differences were observed for any of the indices (Additional file 1).

### Altered correlation network of the microbiome in prion diseases

**Figure 3:**
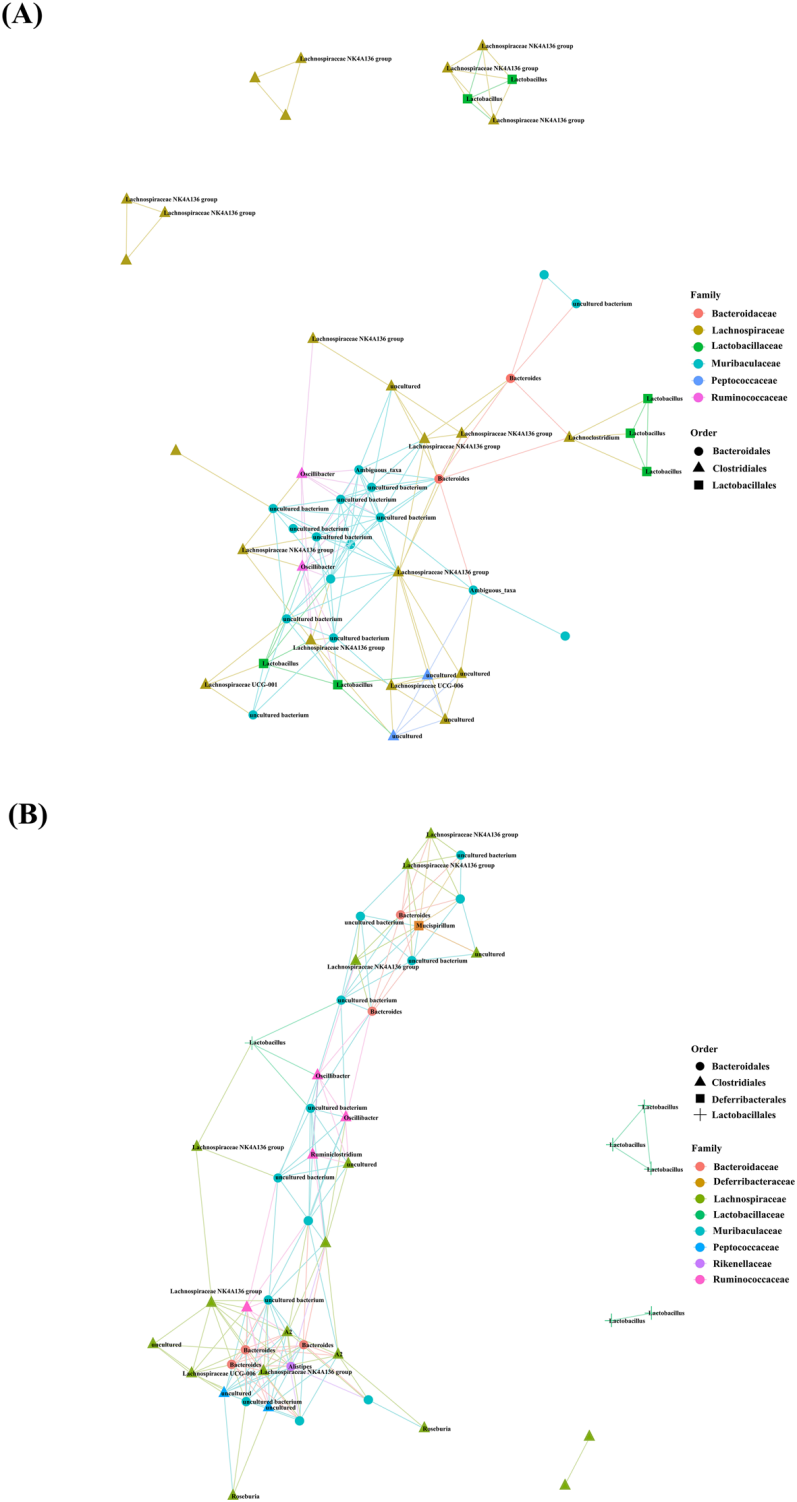
**Correlation network analyses of the top 50 abundant taxa for control mice and prion-infected mice.** **A** Correlation network analysis for control mice. **B** Correlation network analysis for prion-infected mice. The colors indicate family. The shapes indicate order.

We performed a correlation network analysis to assess the impact of differentially abundant microbiomes induced by prion diseases (Figure 3). Notably, the overall shape of the correlation network of prion-infected mice showed a sharp and distorted shape out of the center compared with that of control mice. In addition, the density of the correlation network of prion-infected mice was high compared with that of control mice.

### Differentially enriched Kyoto Encyclopedia of Genes and Genomes (KEGG) pathways between prion-infected and control mice

**Figure 4:**
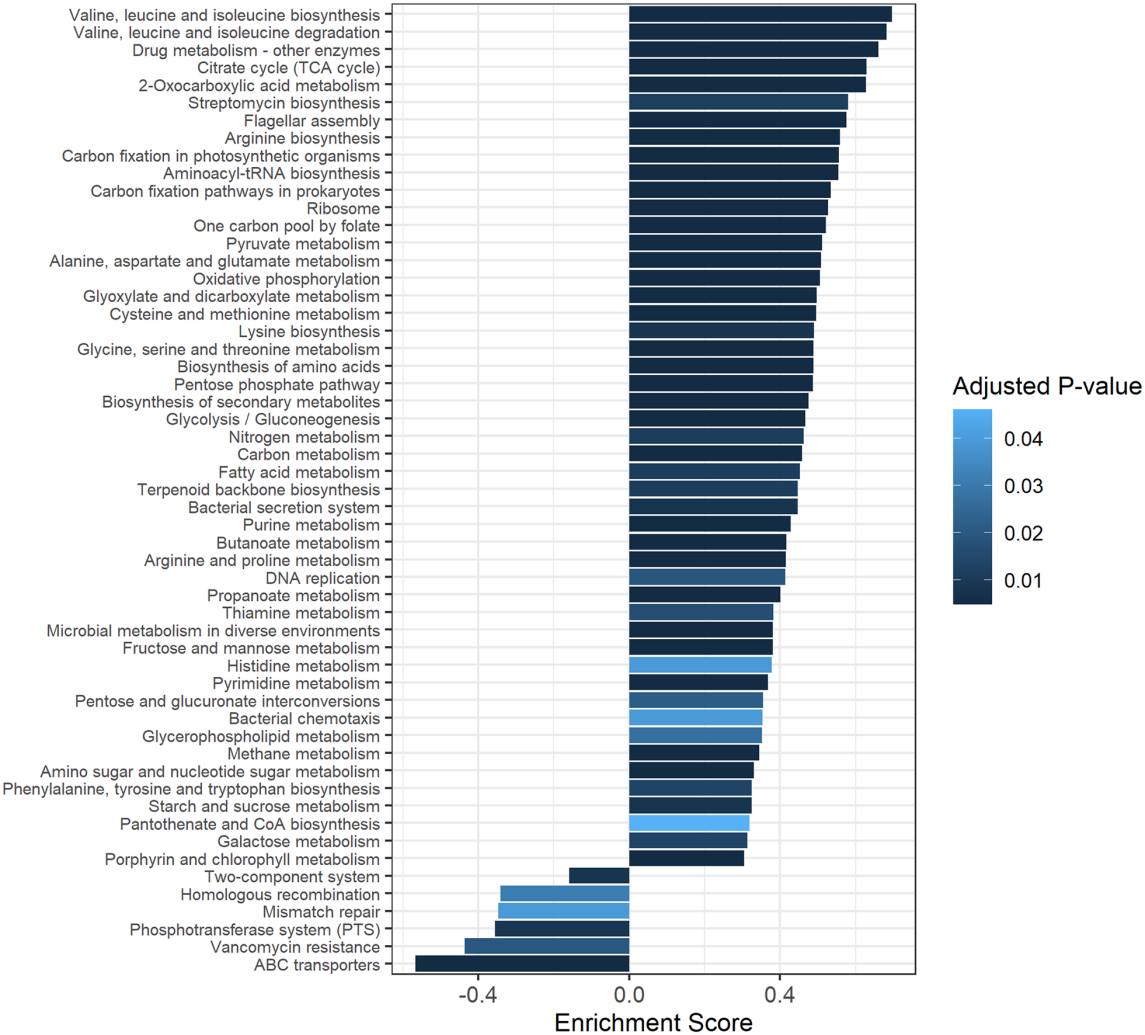
**Differentially enriched KEGG pathways between prion-infected and control mice.** The pathways with adjusted *P* < 0.05 in gene set enrichment analysis (GSEA) were considered significantly enriched. Enrichment scores > 0: enriched in control mice; < 0: enriched in prion-infected mice.

To identify the effect of differentially abundant taxa between prion-infected and control mice, we investigated the functional components using the KEGG pathway database (Figure 4). A total of 55 enriched signaling pathways were identified. In brief, 49 KEGG pathways were upregulated in control mice. The pathway with the highest enrichment score was “Valine, leucine, and isoleucine biosynthesis", followed by “Valine, leucine, and isoleucine degradation", “Drug metabolism—other enzymes", “Citrate cycle (TCA cycle)", “2-oxocarboxylic acid metabolism", and “streptomycin biosynthesis”. In addition, six KEGG pathways were upregulated in prion-infected mice. The pathway with the highest enrichment score was “ABC transporters", followed by “vancomycin resistance", “phosphotransferase system (PTS)", “mismatch repair", “homologous recombination”, and “two-component system".

### Protective effects of EGCG, a potent microbiome changer, in prion-infected mice

**Figure 5:**
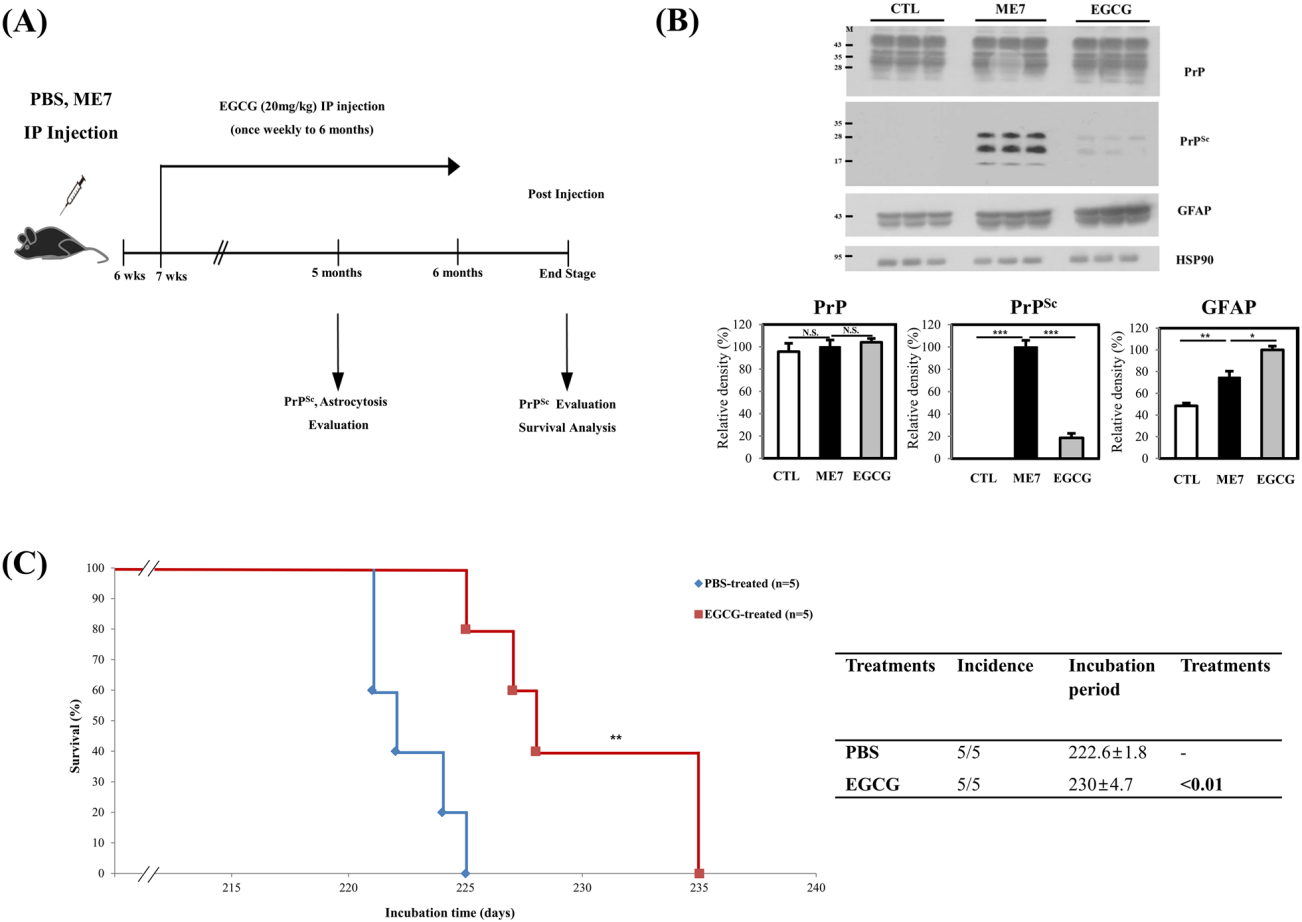
**Evaluation of the protective effects of epigallocatechin-3-gallate (EGCG) in prion-infected mice.** **A** Experimental overview of the evaluation of the effects of EGCG in prion-infected mice. **B** Evaluation of the protective effects of EGCG in prion-infected mice at 5 months post injection. The upper panel indicates western blotting results. The lower panel indicates the quantification of the expression levels of PrP^Sc^ and astrocytosis-related protein normalized to the HSP90 level. CTL: PBS-inoculated mice, *n* = 3; ME7: ME7 scrapie-inoculated mice, *n* = 3; EGCG: EGCG-treated ME7 scrapie-inoculated mice, *n* = 3. **C** Survival analysis in PBS-treated ME7 scrapie-inoculated mice (*n* = 5) and EGCG-treated ME7 scrapie-inoculated mice (*n* = 5). The left panel shows the survival ratio versus days post- inoculation. The right panel indicates the summary of Kaplan‒Meier survival analysis results. Statistical significance was calculated by the log-rank test. ** indicates *P* < 0.01.

The experimental design is shown in Figure 5A. To estimate the protective effects of EGCG against prion disease, we injected the ME7 scrapie strain into 6-week-old mice, treated them with PBS and EGCG (20 mg/kg) weekly, and sacrificed and collected brains to evaluate PrP^Sc^ accumulation and astrocytosis at 5 months post injection (Figure 5B). Notably, PrP^Sc^ accumulation was significantly decreased in EGCG-treated prion-infected mice compared with PBS-treated prion-infected mice at 5 months post injection. However, the astrocyte marker GFAP was upregulated in EGCG-treated prion-infected mice compared with PBS-treated prion-infected mice. We conducted a survival analysis to evaluate the protective effects of EGCG (Figure 5C). Notably, EGCG (230 ± 4.7 days)-treated prion-infected mice showed significantly prolonged survival time compared with PBS-treated prion-infected mice (222.6 ± 1.8 days). We also evaluated PrP^Sc^ accumulation in the brains of EGCG-treated prion-infected mice at the end stage of prion disease, and PrP^Sc^ was detected in those mice (Additional file 2).

## Discussion

In the present study, we identified significant alteration of the microbiome in intraperitoneally prion-infected mice. We observed a total of 14 differentially abundant taxa between prion-infected and control mice. Further analysis of the relationship between each microbiome and prion diseases is highly desirable. To interpret the effect of prion disease on the microbiome, we performed KEGG pathway analysis. Notably, “ABC transporters", “vancomycin resistance", “phosphotransferase system (PTS)", “mismatch repair", “homologous recombination", and “two-component system” were upregulated in the prion-infected mouse-derived microbiome. “ABC transporters", “phosphotransferase system (PTS)", “mismatch repair", “homologous recombination", and “two-component system” are associated with DNA repair-related pathways [26], indicating that prion diseases may induce DNA damage in the microbiome. Further analysis of the effect of differentially abundant taxa in microbiomes and their derivatives on prion diseases is needed in the future.

To validate the association between the microbiome and prion diseases, we evaluated the protective effect of EGCG in prion-infected mice. EGCG is a prevalidated potent microbiome changer and it ameliorates experimental colitis [22]. Notably, in the 5-month group, we observed a significant decrease in PrP^Sc^ and prolonged survival time in EGCG-treated prion-infected mice, while astrogliosis was increased. This paradoxical increase in astrogliosis, despite lower prion burden, may indicate that astroglial activation is not solely dependent on PrP^Sc^ accumulation. EGCG-induced shifts in gut microbiota could alter peripheral immune tone, leading to increased astrocytic activation through systemic cytokine signaling. In addition, EGCG may enhance astrocytic metabolic activity or trigger neuroprotective responses, in which reactive astrocytes proliferate to maintain homeostasis and repair neuronal damage, even in the context of reduced prion load. The recovery of PrP^Sc^ levels observed at the end stage is likely attributable to the prolonged incubation period and the cessation of treatment, rather than a reversal of EGCG’s effects during the earlier phase. This result indicates that prion diseases are strongly related to the microbiome and that the microbiome is an effective drug target for prion diseases. Further development related to the microbiome is recommended in the future. However, since the microbiomes influence each other and are very complex, there is a very high risk of selecting a specific microorganism group for the drug; thus, a careful approach is needed.

Recent studies have reported that the microbiome is related to the pathophysiology of prion, Alzheimer’s, and Parkinson’s diseases [20, 27–30]. Previous studies used the RML strain and employed intracerebral injection for disease induction. Owing to differences in the prion strain used and the route of administration (direct intracerebral injection), the altered microbiota observed in previous studies differed from those identified in the present study. Nevertheless, both studies clearly demonstrated that prion infection induces significant changes in the gut microbiota, underscoring the importance of exploring how microbiota modulation may impact brain pathology. A differentially abundant microbiome was identified between healthy and Alzheimer’s disease animal models, and transfer of the healthy microbiome ameliorated Alzheimer’s disease in the animal model [28]. Since the clear association of the microbiome with prion diseases has been verified in the present study, further studies on the pharmacological efficacy of fecal transplantation are highly desirable in the future. Although the present study did not include microbiome profiling of the ME7 + EGCG group, future studies will aim to investigate the microbiome-related effects of EGCG treatment in the ME7 prion disease model to provide a more comprehensive understanding of its therapeutic potential.

In conclusion, we identified a total of 14 differentially abundant taxa between prion-infected and control mice. In addition, we observed that prion diseases induced an altered microbiome network and upregulation of DNA repair-related pathways. We found a protective effect of the microbiome changer EGCG in prion-infected mice. To the best of our knowledge, this is the first report of a strong association between the microbiome and prion diseases.

## Supplementary Information

**Additional file 1. Comparison of alpha diversity between prion-infected (ME7) and healthy control (CTL) groups.** Alpha diversity indices, including Observed OTUs, Shannon diversity, Pielou’s evenness, and Faith’s phylogenetic diversity, were compared using the Wilcoxon rank-sum test. N.S.: Not significant.

**Additional file 2. Western blotting detection of PrPScin EGCG‑treated ME7 scrapie‑inoculated mice at the end stage.** PK: proteinase K; -: Proteinase K-untreated lane; +: Proteinase K-treated lane.

**Additional file 3 Uncropped Gels and Blots images.**

## Data Availability

The data have been deposited in the NCBI Sequence Read Archive (SRA) under accession no. PRJNA1310243.
