# Supplementary material for: Identification of an altered gut microbiome and the protective effect of microbiome changer in prion diseases
Source: Vet Res. 2026 Jan 17;57:31. doi: 10.1186/s13567-025-01699-2 (PMC12895704; doi:10.1186/s13567-025-01699-2)
Supplement: Supplementary file 1 — Additional file 1. Comparison of alpha diversity between prion-infected (ME7) and healthy control (CTL) groups. Alpha diversity indices, including Observed OTUs, Shannon diversity, Pielou’s evenness, and Faith’s phylogenetic diversity, were compared using the Wilcoxon rank-sum test. N.S.: Not significant. [file 13567_2025_1699_MOESM1_ESM.docx]

**Identification of an altered gut microbiome and the protective effect of microbiome changer in prion diseases**

Yong-Chan Kim^1, 2, #^, Sae-Young Won^3,4, #^, Byung-Hoon Jeong^3,4 *^

*^1^Department of Biological Sciences, Andong National University, Andong 36729, Republic of Korea.*

*^2^School of Life Sciences and Biotechnology, Gyeongkuk National University, Andong 36729, Republic of Korea.*

*^3^Korea Zoonosis Research Institute, Jeonbuk National University, Iksan, Jeonbuk 54531, Republic of Korea*

*^4^Department of Bioactive Material Sciences* *and Institute for Molecular Biology and Genetics, Jeonbuk National University, Jeonju, Jeonbuk 54896, Republic of Korea*

*** Corresponding author:**

Byung-Hoon Jeong, Ph.D.

Korea Zoonosis Research Institute, Jeonbuk National University, 820-120

Hana-ro, Iksan, Jeonbuk, Republic of Korea

TEL: 82-63-900-4040, FAX: 82-63-900-4012, E-mail: bhjeong@jbnu.ac.kr

^#^These authors contributed equally to this work.

**
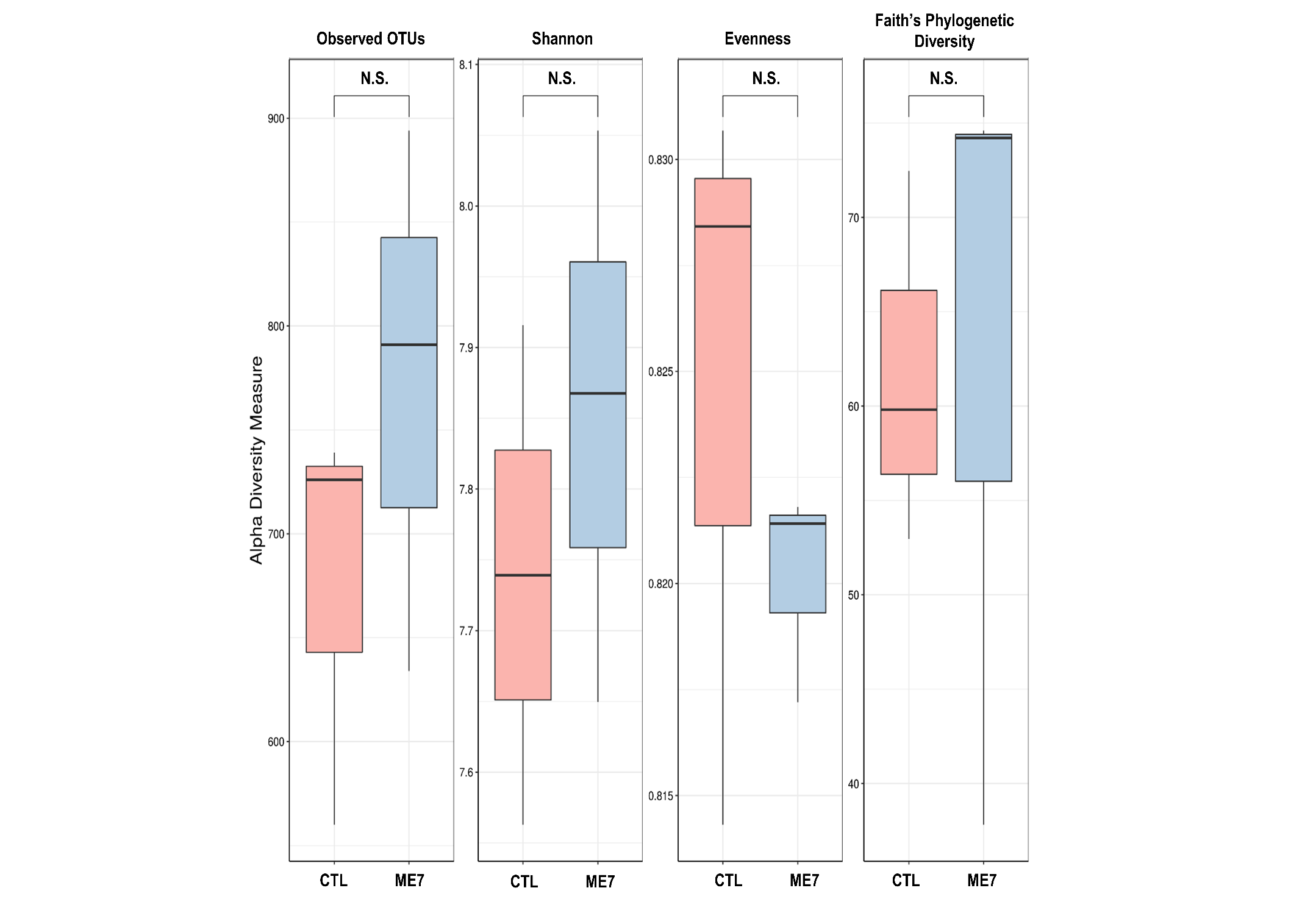
**

**Additional file 1**

Comparison of alpha diversity between prion-infected (ME7) and healthy control (CTL) groups. Alpha diversity indices, including Observed OTUs, Shannon diversity, Pielou’s evenness, and Faith’s phylogenetic diversity, were compared using the Wilcoxon rank-sum test. N.S.: Not significant.
