# Supplementary figures and images for: Identification of an altered gut microbiome and the protective effect of microbiome changer in prion diseases
Source: Vet Res. 2026 Jan 17;57:31. doi: 10.1186/s13567-025-01699-2 (PMC12895704; doi:10.1186/s13567-025-01699-2)

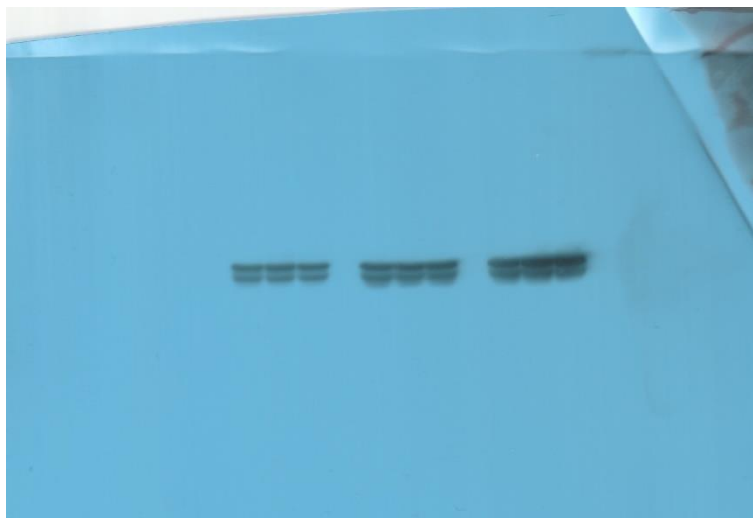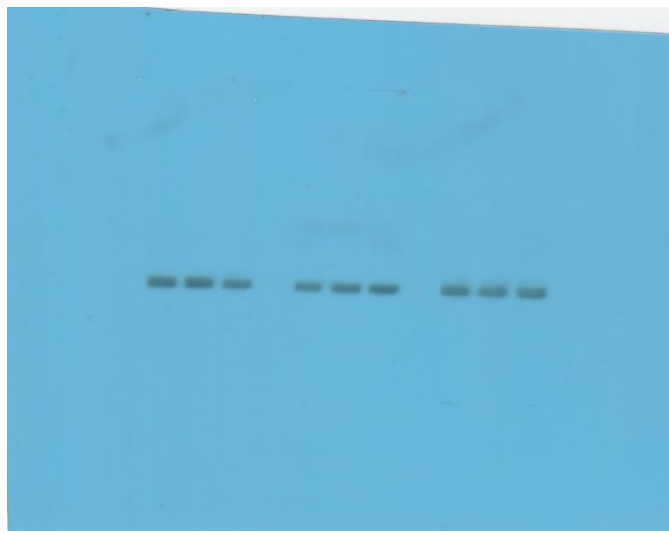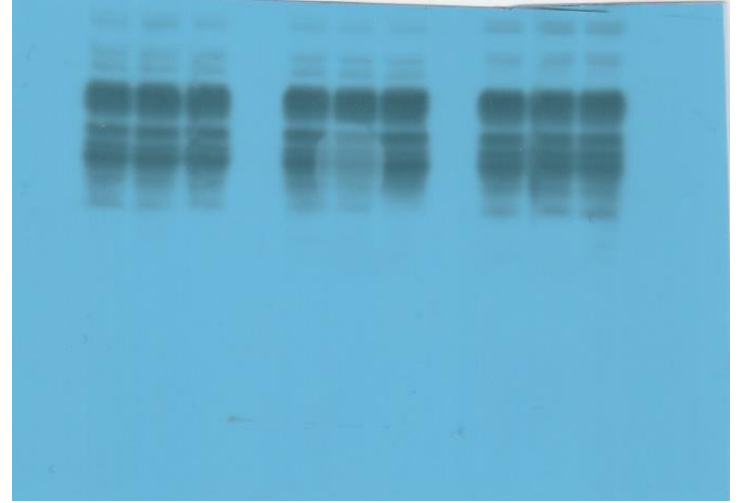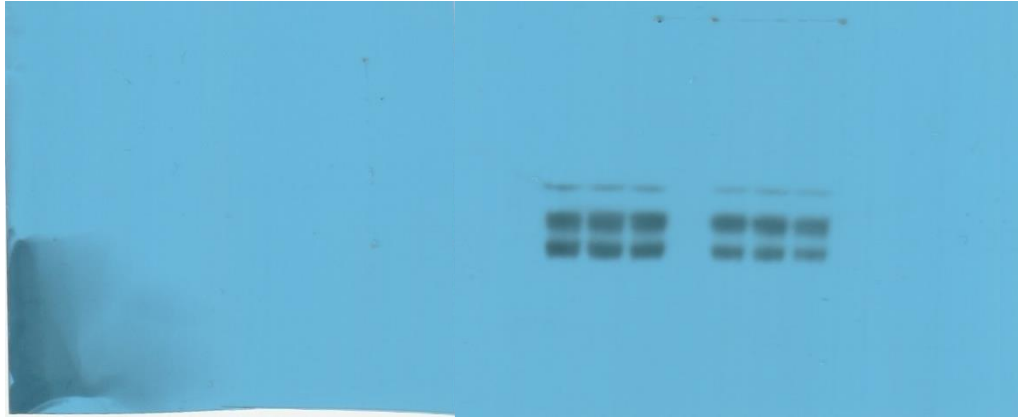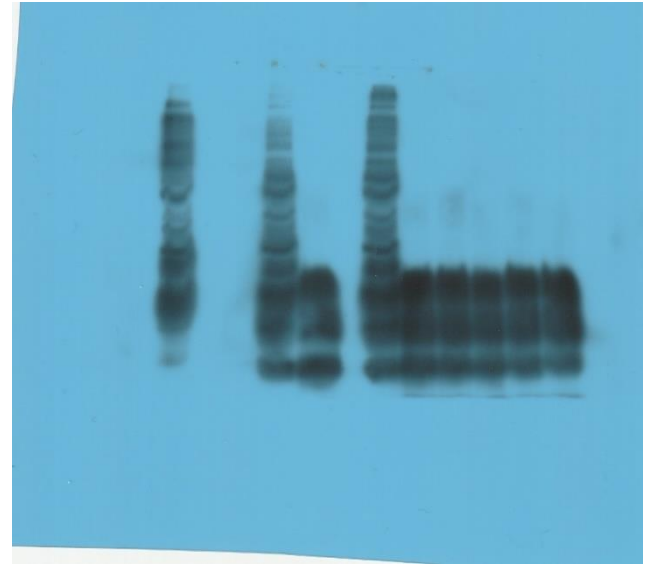

Supplement: Supplementary file 3 — Additional file 3 Uncropped Gels and Blots images. [file 13567_2025_1699_MOESM3_ESM.pdf]
